# Tulathromycin treatment does not affect bacterial dissemination or clearance of *Brucella melitensis* 16M following experimental infection of goats

**DOI:** 10.1371/journal.pone.0226242

**Published:** 2019-12-10

**Authors:** Paola M. Boggiatto, Steven C. Olsen

**Affiliations:** Infectious Bacterial Diseases Research Unit, National Animal Disease Center, Agricultural Research Service, U.S. Department of Agriculture, Ames, Iowa, United States of America; East Carolina University Brody School of Medicine, UNITED STATES

## Abstract

Brucellosis in sheep and goats, a zoonotic disease primarily associated with *Brucella melitensis* infections, causes significant economic losses and public health concerns worldwide. Although control measures are effective, economic limitations and nomadic lifestyles may limit vaccination coverage, and test and removal policies may not be feasible. In this study, we evaluated the effects of therapy with a long acting antimicrobial tulathromycin on the pathogenesis of brucellosis. Thirty-five goats were randomly assigned for experimental infection with *B*. *melitensis* strain 16M while open or during mid-gestation. Approximately half of the animals in each group were then treated with tulathromycin and subsequently assessed for the development of humoral responses to infection, clinical presentation, and bacterial dissemination and colonization. All animals, regardless of treatment group were successfully challenged with *B*. *melitensis* 16M demonstrated by bacterial recovery from conjunctival swabs and development of positive antibody titers. In goats infected while open, no animals aborted and *Brucella* was recovered from only one animal in tulathromycin-treated and one animal from the untreated group. Tulathromycin treatment of pregnant goats did not prevent abortion nor did it reduce bacterial dissemination, colonization, or shedding. Our data suggests that treatment of goats in mid-gestation with tulathromycin at the labeled dose does not influence disease pathogenesis or tissue colonization after experimental *B*. *melitensis* challenge.

## Introduction

Brucellosis is an important zoonosis caused by a gram-negative bacterium that is reemerging in many parts of the world. In natural hosts, brucellosis is most commonly associated with reproductive losses and infertility, but can also cause arthritis, mastitis, and other pathologic lesions [1]. *Brucella melitensis*, *B*. *suis*, and *B*. *abortus* are the most important zoonotic species within the *Brucella* genus with *B*. *melitensis* having the greatest virulence in humans. Although most *Brucella* species have the ability to infect a number of hosts, each has a preferred animal host. Goat and sheep are the preferred host for *B*. *melitensis*.

Many countries have programs to control or eradicate zoonotic brucellae (*B*. *abortus*, *B*. *melitensis* and *B*. *suis*) from domestic livestock primarily due to the benefits for human public health. Multiple studies have demonstrated that controlling brucellosis in animal reservoirs is the most cost-effective mechanism for addressing human brucellosis [2, 3]. Regulatory efforts can include sanitation programs to prevent disease transmission, vaccination programs to reduce herd susceptibility, and test and removal programs to eliminate animals infected with brucellosis. Vaccination programs alone have not been successful in eradicating animal brucellosis, and currently there are no human vaccines available. *B*. *melitensis* Rev.1 vaccine has been widely used in certain developed countries, however, this vaccine induces abortions in pregnant animals, it is virulent to humans and it induces anti-*Brucella* titers that interfere with diagnostic test (reviewed in [4]). In addition, mass vaccination practices of infected herds help reduce disease prevalence but do not fully resolve public health concerns. Therefore, the use of alternative control practices, such as antimicrobial therapy combined with selective culling and/or vaccination might help reduce disease burden and zoonotic potential.

Antibiotic treatment in veterinary species to address *Brucella* infections has had limited efficacy and has not been incorporated into regulatory programs. Historically, failure of antibiotic treatment (i.e. continued *Brucella* shedding in udder secretions and *Brucella* persistence in tissues) have been shown to result from improper antimicrobial choice [5–7], improper dose [8–11], or inappropriate duration of treatment [8–11]. Various therapeutic regimens utilizing oxytetracycline (OTC), long-acting OTC alone or in combination with streptomycin (ST) have shown some success in eliminating clinical signs and brucellae shedding in multiple species, however, these regimens fail to achieve complete cure [9, 11–12]. Not unexpectedly, therapeutic regimens with longer treatment periods tend to be more successful in clearing *Brucella*. However, longer treatments have the potential to cause both local and systemic reactions, increased withdrawal times, greater expense, induce antibiotic resistance, and ultimately are not always effective.

The failure of most antibiotic regimes against *Brucella* can be attributed, in part, to an inability to reach the intracellular environment where the bacteria localize. In order to enhance intracellular uptake of antimicrobials, [10] encapsulated ST or ST/LA-OTC within liposomes. Liposomes are readily taken up by macrophages, and therefore provide a mechanism to increase intracellular concentrations of these antibiotics, especially within these target cells. However, treatment of cows naturally-infected with *B*. *abortus* with liposome encapsulated antibiotics did not result in complete clearance of organisms from tissues nor did it prevent shedding of *Brucella* within udder secretions [10]. Nevertheless, intracellular delivery of antibiotics has several potential benefits including increasing antimicrobial concentrations within the *Brucella* intracellular niche, prolonging antibiotic activity, and potentially reducing the number of treatments required.

Macrolide antibiotics are a large family of protein synthesis inhibitors that act by binding to the bacterial 50s ribosomal subunit. Tulathromycin is a semi-synthetic macrolide and belongs to a subclass of macrolides known as the triamilides [13]. It is characterized by a rapid rate of absorption, large systemic availability (approximately 90%) following intramuscular or subcutaneous administration, and a long half-life ranging from 60–140 hours in domestic species (reviewed in [14]). Currently, tulathromycin is approved in the United States for the treatment and control of bovine respiratory disease (BRD) and the treatment of respiratory conditions in swine [15]. Studies of tulathromycin in goats have been performed and have demonstrated similar pharmacokinetics [16] and tissue distribution and residue levels [17] to those reported in cattle at the recommended 2.5 mg/kg subcutaneous (SC) injection dose and extra-label use of tulathromycin in goats is common for the treatment of respiratory conditions.

Macrolide antibiotics have been previously tested for their effectiveness against *Brucella* infection. Dieste-Perez *et al*. tested the *in vitro* susceptibility of *B*. *suis* reference and field strains to macrolide antibiotics (tulathromycin and tildiprosin), demonstrating MIC_90_ values that ranged from 0.01–0.25 ug/ml [18]. However, usage of macrolide antibiotics alone for the treatment of brucellosis has not been tested in natural hosts. Therefore, given the preferred intracellular distribution of macrolide antibiotics, extended half-life *in vivo*, and previous data demonstrating *in vitro* and *in vivo Brucella* susceptibility, we wanted to test the effects of treatment with the macrolide antibiotic, tulathromycin (Draxxin^®^, Zoetis), on goats experimentally-challenged with *B*. *melitensis* 16M.

## Materials and methods

### Bacterial cultures

*B*. *melitensis* strain 16M was obtained from the National Animal Disease Center (Ames, IA) culture collection. Frozen stock cultures used for experimental infection or reagent preparation were propagated on tryptose agar (Difco Laboratories, Detroit, MI) containing 5% bovine sera (TSA) for 72 hours at 37°C and 5% CO_2_. Bacteria were harvested via resuspension in phosphate-buffered saline (PBS), and bacterial concentrations were determined via measurement of the optical density at 600 nm with a spectrophotometer adjusted based on a OD_600_/CFU calibration curve. Final concentrations of live bacteria used for animal challenges were determined by serial dilution and standard plate counts on TSA. For use in serology assays, strain 16M bacteria were grown on TSA for 48 hours at 37°C and resuspended in PBS and heat inactivated at 60°C. Bacterial concentrations within the suspension were determined by standard plate counts prior to heat inactivation. Following confirmation of inactivation by microbiological culture, aliquots of the culture suspension were stored at -80°C until use. All culture manipulations were performed in a certified biosafety cabinet in a Select Agent-registered space, using biosafety level (BSL)-3-level precautions.

### Experimental challenge of goats and antibiotic treatment

Thirty-five nannies of approximately 1 to 3 years of age were obtained from the resident brucellosis-free herd on campus. The group contained Toggenburg, Alpine, Saanen, and Nubian breeds. After onsite acclimation for two weeks, animals were randomly assigned into two experimental groups: 1) infection while open (n = 16) and 2) infection during mid-gestation (n = 18).

Animals assigned into the infection while open were moved into an agricultural biosafety level (AgBSL)-3 facility at the National Animal Disease Center (NADC) in Ames, IA and allowed to acclimate for an additional two weeks prior to intraconjunctival challenge with 10^7^ colony forming units (CFU) of *B*. *melitensis* strain 16M. At 5 weeks after challenge, some nannies (n = 8) were randomly chosen to receive antibiotic treatment. Nannies in the antibiotic treatment group received 2.4 mg/kg of tulathromycin (Draxxin^®^, Zoetis) intramuscularly (IM), at 4 and 6 weeks after experimental infection. Remaining animals (n = 8) were not administered the antibiotic treatment. Blood was obtained from the jugular vein at 2, 4, 8, 13, 23, and 27 weeks after challenge for serology and/or isolation of peripheral blood mononuclear cells. Male goats were placed with treated and untreated goats in AgBSL-3 housing at 10 weeks after challenge and co-housed with nannies until the termination of the project. Animals were maintained under AgBSL-3 conditions until parturition or euthanasia at 37 or 38 weeks after challenge.

Animals assigned to be infected during pregnancy were pasture bred in the fall and pregnancy confirmed by measurement of pregnancy-specific protein B in serum (MidWest Veterinary Associates, Centerville, IA). Following confirmation of pregnancy, animals were moved to a AgBSL-3 containment facility. After acclimation for two weeks, animals were intra-conjunctivally challenged at mid-gestation with approximately 10^7^ CFU of *B*. *melitensis* strain 16M. Three weeks post-challenge, half of goats (n = 9) were randomly chosen to receive antibiotic treatment. Nannies in the antibiotic treatment group received 2.4 mg/kg of tulathromycin (Draxxin^®^, Zoetis) intramuscularly (IM), once. The remaining nannies (n = 9) were left untreated. Blood was obtained from the jugular vein at 0 and 4 weeks post-challenge for serology. Animals were maintained under AgBSL-3 conditions until abortion or parturition.

Large animal isolation facilities were operated under guidelines approved by the United States Department of Agriculture/Agricultural Research Service (USDA/ARS). All animal studies were performed under approval from the Institutional Animal Care and Use Committee (IACUC) at the NADC.

### Confirmation of experimental challenge with *B*. *melitensis* 16M

Experimental challenge was confirmed via two methods: recovery of the challenge strain and seroconversion. Conjunctival swabs were taken from each nannie at 5 days post-challenge, plated onto Kuzdas and Morse (KM) media [19], and incubated at 37°C in 5% CO_2_ for 7 days to verify the presence of *B*. *melitensis* by microbiologic technqiues [20]. Isolates were confirmed as *Brucella* via polymerase chain reaction (PCR) based on colony morphology and using *Brucella*-specific primers for *omp2a* [21]. For serology, blood was obtained from the jugular vein of goats infected while open at 0, 2, 4, 8, 12, 23, and 37 weeks and in goats infected during pregnancy at 0 and 4 weeks after experimental challenge. Antibody responses were evaluated using a standard tube agglutination test [20].

### *B*. *melitensis* 16M-specific enzyme-linked immunosorbent assay

Antibody (IgG) responses against *B*. *melitensis* 16M were determined by a previously described ELISA [21] using methanol-killed *B*. *melitensis* as antigen and an anti-goat IgG horseradish peroxidase (HRP)-conjugated secondary antibody (Jackson ImmunoResearch, West Grove, PA).

### Necropsy and tissue processing

After abortion or parturition, nannies were euthanized by intravenous injection of sodium pentobarbitol (Sleepaway, Ft. Dodge Labs, Ft. Dodge, IA, USA). Maternal samples obtained at necropsy after experimental challenge for microbiologic evaluation included: lymphatic tissues (bronchial, hepatic, internal iliac, mandibular, parotid, prescapular, retropharyngeal, and supramammary), lung, liver, spleen, placentome or uterus, mammary gland (both glands), milk, blood, vaginal swabs, and conjunctival swabs. Kids born live were humanely euthanized by intravenous injection of sodium pentobarbitol. Necropsies of euthanized kids and fetuses were performed and tissues collected included: lung, liver, spleen, bronchial lymph node, blood, and gastric contents.

Tissue samples for bacterial enumeration were processed as previously reported [22]. Briefly, approximately 1 g of tissue sample was individually ground in 2 ml of PBS (pH = 7.2) using glass Dounce homogenizers, serially diluted, plated onto KM plates. Vaginal and conjunctival swabs were streaked directly onto KM plates. Blood samples were mixed 1:1 (vol:vol) with tryptose broth (Difco Laboratories, Detroit, MI) containing 1% sodium citrate. After plating on KM plates, blood samples were held at 4°C for 24 hours, then incubated at 37°C and 5% CO_2_ with aliquots plated onto media at 7, 14, 21 and 28 days. All KM plates were incubated at 37°C and 5% CO_2_ for up to 7 days. Isolates were identified as *Brucella* on the basis of colony morphology, growth characteristics, and a *Brucella*-specific PCR assay [20, 21].

### Statistical analysis

Colonization (CFU/gm) data was converted to a logarithm for analysis. Standard tube agglutination data which was negative on the first dilution and colonization data in which no recovery was made was converted to 1 for logarithmic conversion. Means were separated by a least square means procedure (*p* ≤ 0.05). Fisher’s exact test was used to evaluate differences in incidence of infection between treatment groups.

## Results

### *B*. *melitensis* 16M inoculum and confirmation of infection

Separate challenge inocula were prepared as to deliver 10^7^ CFU of *B*. *melitensis* 16M via the intraconjunctival route. Standard plate counts of each challenge inoculum indicated that nannies infected while open received 1.5 x 10^8^ CFU of *B*. *melitensis* 16M while goats infected while pregnant were challenged with 4.5 x 10^6^ CFU of *B*. *melitensis* 16M. *Brucella* was recovered from conjunctival swabs from all goats at 5 days after challenge, indicating successful infection of all animals.

### Tulathromycin treatment does not affect humoral responses to *B*. *melitensis* 16M in open or pregnant goats

**Fig 1:**
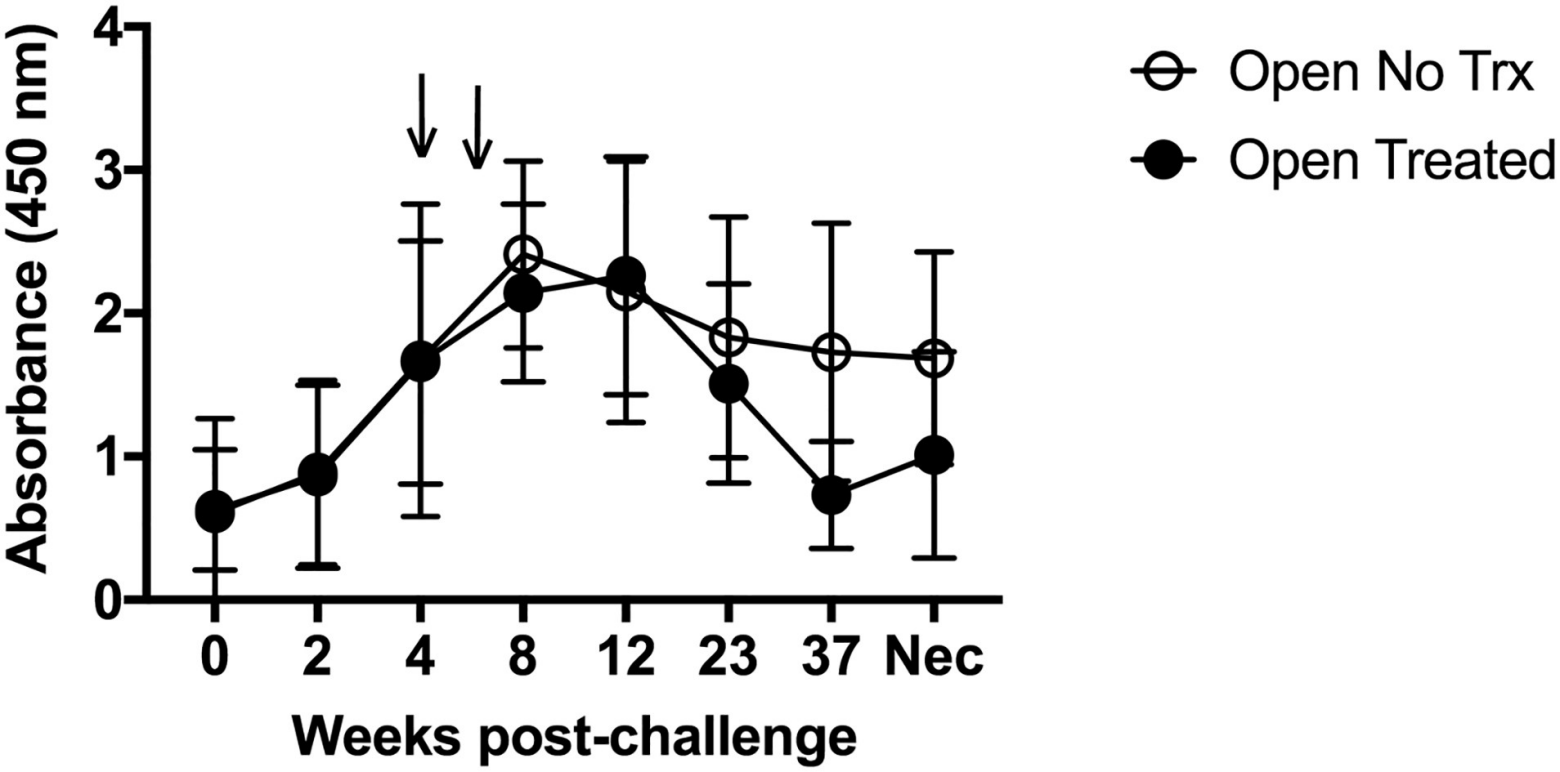
Assessment of antigen-specific IgG responses in serum following challenge of goats with *B*. *melitensis* 16M. Time course of *B*. *melitensis* 16M-specific total IgG in serum of goats infected with *B*. *melitensis* 16M that were either treated (black circles) or not treated (open circles) with tulathromycin. Arrows denote timing of tulathromycin administration, 4 and 6 weeks post-challenge.

In the open group, all nannies in this group were negative for serologic responses to *B*. *melitensis* 16M via the standard tube agglutination test (STAT) (Table 1) prior to challenge. However, by four weeks post-challenge, 11 out of 16 (68%) were positive on the standard tube agglutination test, and by 8 weeks post-challenge, 15 of 16 (93%) were positive (Table 1). All nannies demonstrated increased humoral responses to *Brucella* on the ELISA assay, peaking around 8 weeks post-challenge and declining over time (Fig 1). Antibiotic treatment did not influence humoral resposnes as no differences (*p* ≥ 0.05) in responses were observed between treated and non-treated goats (Fig 1). Collected data confirmed the experimental challenge was successful and suggested that tulathromycin treatment did not influence the humoral response of goats to *B*. *melitensis* 16M infection.

**Table 1:** Standard agglutination tube data for open animals prior to and post-*B*. *melitensis* 16M challenge.

Goat #	Treatment group	Weeks post-challenge			
		0	2	4	8
1	No Treatment	N25	N25	N25	100
3		N25	400	N25	400
6		N25	400	400	200
8		N25	400	100	100
11		N25	N25	100	400
14		N25	50	N25	100
31		N25	N25	>400	25
32		N25	>400	N25	25
4	Tulathromycin	N25	N25	400	50
9		N25	25	25	50
10		N25	N25	400	50
15		N25	N25	50	50
24		N25	400	N25	N25
25		N25	N25	400	100
26		N25	100	400	100
27		N25	400	400	400

Values are indictive of the highest dilution performed at which animals were positive.

N25: negative at the lowest dilution performed, 1:25.

**Fig 2:**
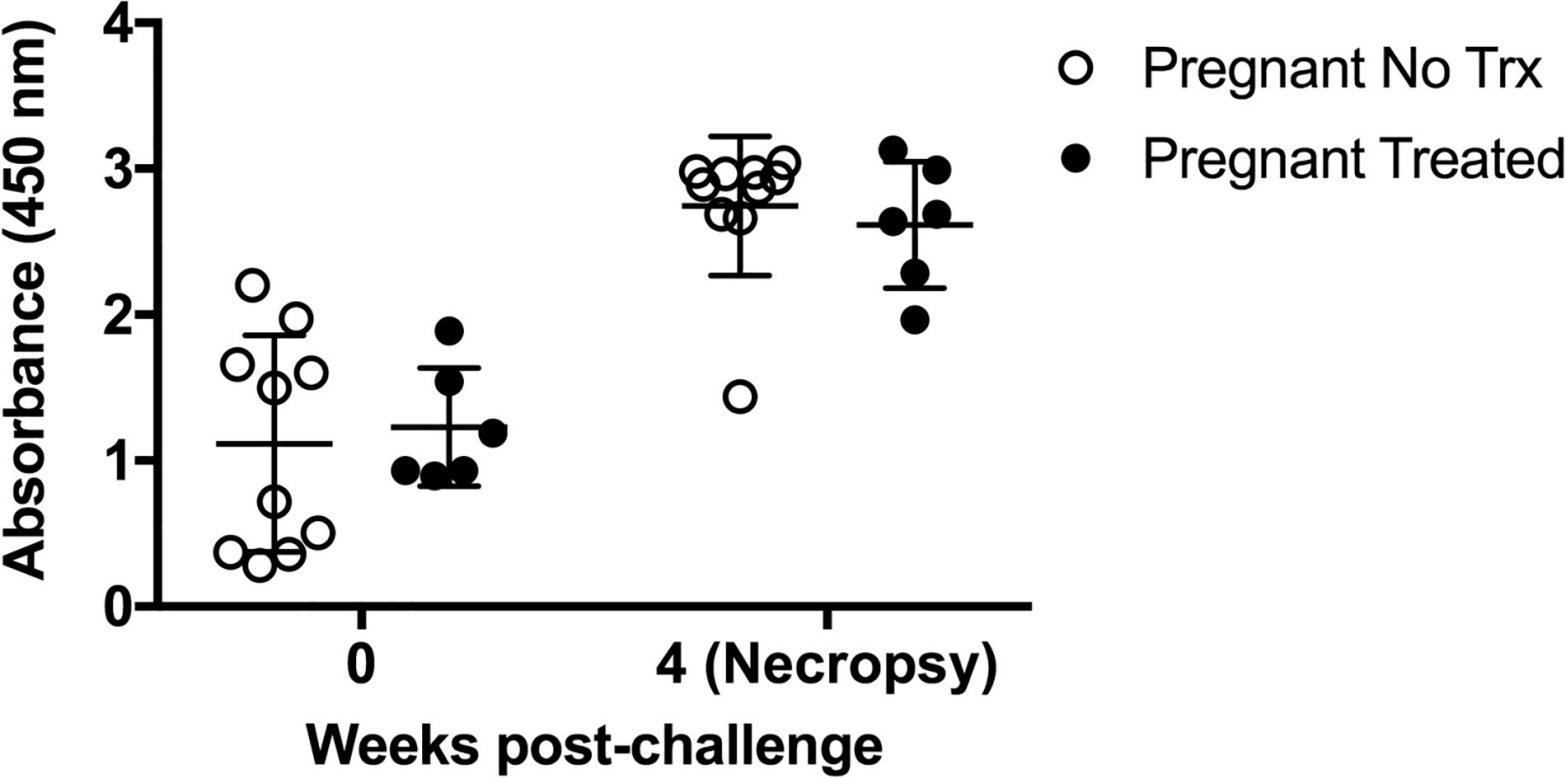
Antigen-specific IgG responses of pregnant goats following *B*. *melitensis* 16M challenge. Shown are IgG titers prior to and at 4 weeks post-challenge in pregnant goats either treated (black circles) or not treated (open circles) with tulathromycin.

In goats infected in mid-gestation, one goat in the non-treated group had a titer in the suspect range on the standard tube agglutination test whereas all others were negative prior to experimental challenge (Table 2). However, all animals had increased humoral responses on the STAT (Table 2) and ELISA at 4 weeks post-challenge (Fig 2). Tulathromycin treatment had no effect (*p* ≥ 0.05) on antibody responses of challenge animals.

**Table 2:** Standard agglutination tube data for pregnant goats prior to and post-*B*. *melitensis* 16M challenge.

Ear Tag #	Treatment group	Weeks post challenge	
		0	4 (Nec)
2	No treatment	N25	100
5		N25	400
7		N25	400
16		N25	400
17		N25	100
18		100	25
19		N25	50
20		N25	400
21		N25	400
33	Tulathromycin	N25	>400
34		N25	>400
35		N25	>400
13		N25	400
22		N25	400
23		N25	400
28		N25	400
29		N25	400
30		N25	25

Values are indictive of the highest dilution performed at which animals were positive.

N25: negative at the lowest dilution performed, 1:25.

### Tulathromycin treatment does not influence reproductive efficiency or *B*. melitensis 16M infection in open goats

Ten weeks after challenge, or 3 weeks after the last tulathromycin treatment, nannies in the open treatment group were cohoused with bucks for natural breeding while in confinement. Photoperiods within the rooms were not changed. No abortions were observed after breeding in animals that were infected while open. At the end of the study, 6/9 (66.7%) nannies in the tulathromycin-treated group were found to be pregnant, whereas 4/8 (50%) in the non-treated group were pregnant (Table 3). Although limited, our data suggests that tulathromycin treatment did not influence reproductive efficiency.

**Table 3:** Pregnancy detection and pregnancy rate in goats infected while open and either Tulathromycin-treated or non-treated.

Time of infection	Treatment	Live Kids	Abortions	Recovery of *Brucella*
Open	No treatment	6/9 (67%)	0/6 (0%)	1/9 (11%)
Open	Tulathromycin	4/8 (60%)	0/4 (0%)	1/8 (14%)
Pregnant	No treatment	1/9 (11%)	8/9 (89%)	8/9 (88.9%)
Pregnant	Tulathromycin	1/9 (11%)	8/9 (89%)	8/9 (88.9%)

The challenge strain was recovered from conjunctival and vaginal swabs from one tulathromycin-treated group and from lung tissues from one kid in the non-treated group (Table 3). With these exceptions, *Brucella* was not recovered from any other samples obtained at necropsy from this group. Because *Brucella* could only be recovered at necropsy from 2 goats infected while open, tulathromycin treatment did not influence (*p* ≥ 0.05) bacterial colonization in this group.

### Tulathromycin treatment does not affect reproductive outcome or bacterial tissue dissemination of *B*. *melitensis* 16M in pregnant goats

Antibiotic treatment of goats infected during pregnancy did not (*p* ≥ 0.05) influence disease pathogenesis or tissue colonization (Tables 3 and 4). In treated and non-treated goats, 8/9 (88.9%) aborted between 3 and 6 weeks after experimental challenge and the mean time to abortion did not differ (*p* ≥ 0.05) between treatments groups (28.3 ± 1.9 days for non-treated and 31.3 ± 5.7 days for tulathromycin-treated). Although brucellosis is typically associated with expulsion of fresh fetuses or birth of weak kids, aborted fetuses in this study demonstrated moderate to severe autolysis. As shown in Table 4, *Brucella* was recovered from multiple samples from all animals which aborted. However, colonization within tissues (CFU/gm) did not differ (*p* ≥ 0.05) between treated and non-treated groups.

**Table 4:** *B*. *melitensis* tissue colonization and bacterial loads.

	*B*. *melitensis* (CFU/g)	
Tissue	No treatment	Tulathromycin
Parotid LN	2.86 ± 0.49	2.43 ± 0.55
Pre-scapular LN	2.31 ± 0.46	2.71 ± 0.46
Supramammary LN	2.84 ± 0.45	3.19 ± 0.72
Hepatic LN	1.44 ± 0.44	1.91 ± 0.61
Retropharyngeal LN	2.06 ± 0.42	2.78 ± 0.51
Lung	1.92 ± 0.41	1.63 ± 0.54
Liver	1.44 ± 0.32	1.41 ± 0.49
Spleen	1.47 ± 0.43	1.86 ± 0.42
Placentome	5.62 ± 1.46	5.71 ± 1.13
Mammary gland	1.85 ± 0.56	2.32 ± 0.61

Colonization, presented as colony-forming units per gram of tissue (CFU/g), of *B*. *melitensis* 16M in tissues collected at necropsy from pregnant goats not treated or treated with 2.4 mg/kg tulathromycin 3 weeks after experimental infection.

In goats that did not abort, parturition occurred at 85 days (tulathromycin-treated) and 75 days (non-treated). *Brucella* was not recovered from any fetal or maternal tissue obtained at necropsy of animals reaching full-term parturition.

## Discussion

While bovine brucellosis has been eradicated from many countries, small ruminant brucellosis continues to be a major public health and economic burden worldwide. Eradication of small ruminant brucellosis, primarily caused by *B*. *melitensis*, has been difficult because of its prevalence in herds of low-income and/or small production farmers, often nomadic, in developing countries. Although the *B*. *melitensis* Rev-1 vaccine is effective in both sheep and goats (reviewed in [23]), vaccination programs are frequently limited in developing countries due to a lack of resources to implement control programs. In addition, the Rev-1 vaccine can induce serologic responses which interfere with diagnostic testing to detect infection by field strains of *B*. *melitensis*. Alternative control strategies, such as antibiotic treatment alone, or in combination with other methods, might be another approach to reducing the burden of brucellosis.

Historically, antibiotics have not been used to control brucellosis in animal reservoirs, primarily due to cost and lack of effectiveness. Although a combination of oxytetracycline (OTC) and dihydrostreptomycin has been successfully used to treat brucellosis in small ruminants [11, 24], therapeutic regimens are prolonged and expensive, essentially making them unfeasible for use in developing countries. Monotherapy has proven inadequate for treating brucellosis, as relapses in humans are common. This may be due to bacteriostatic effects of commonly used antibiotics, especially tetracyclines, and the intracellular nature of *Brucella* spp. [25]. As new classes of antibiotics become available, specifically long-lasting or extended release antimicrobials, monotherapy may become feasible for addressing brucellosis in natural reservoirs. First generation macrolide antibiotics were proposed as potential agents against brucellosis and initial studies *in vitro* demonstrated promising results [26] against several strains of *Brucella*.

In the current study, we assessed the therapeutic effects of a currently available macrolide antibiotic, tulathromycin (Draxxin^®^), for controlling *B*. *melitensis* infections in its natural host, goats. In nannies infected while pregnant, *B*. *melitensis* 16M was able to establish infection and cause abortions. However, tulathromycin administration did not reduce the incidence of abortions, bacterial loads in tissues, or the potential for shedding. While *in vitro* studies have shown susceptibility of *Brucella* to tulathromycin, the dose administered in the current experiment was not effective in controlling infection. Interestingly, in nannies infected while open, *B*. *melitensis* 16M was able to establish infection, however, regardless of tulathromycin treatment, we did not oberve any abortions nor were we able to recover bacteria from any of these tissues analyzed. These data suggests that the timing of infection (open vs. pregnant) could influence the ability of goats to clear *B*. *melitensis* 16M infections. We have observed in other natural hosts that non-pregnant animals are less susceptible to *Brucella* infections (S. Olsen, personal observation), and this observation is consistent with data from the current study.

Choice of antimicrobial, dose and duration of treatment are important for controlling *Brucella* infections in natural hosts and monotherapies are often unsuccessful at controlling infections. However, tulathromycin with its approval for use in food animals (albeit off-label for goats and sheep) its extended half-life, intracellular-distribution, single administration regimen, and demonstrated *in vitro* susceptibilitiy of *Brucella*, made it an attractive candidate for use in the present study. The therapeutic regiment used here was based on the labeled dose for treatment respiratory infections in cattle and swine. While we did not find tulathromycin to be effective at clearing *B*. *melitensis* 16M from infected pregnant goats with our current regimen, it cannot be excluded that higher treatment doses or other treatment regimens might yield a positive outcome. Next generation antibiotics combined with novel delivery mechanisms may eventually provide an alterantive therapeutic approach for intracellular diseases, such as brucellosis, that will be beneficial for disease control, animal welfare, and human health.
